# Lp(a)-Lowering Agents in Development: A New Era in Tackling the Burden of Cardiovascular Risk?

**DOI:** 10.3390/ph18050753

**Published:** 2025-05-19

**Authors:** Niki Katsiki, Michal Vrablik, Maciej Banach, Ioanna Gouni-Berthold

**Affiliations:** 1 Department of Nutritional Sciences and Dietetics, International Hellenic University, 57400 Thessaloniki, Greece; nikikatsiki@hotmail.com; 2 School of Medicine, European University Cyprus, 2404 Nicosia, Cyprus; 3 Third Department of Medicine, First Faculty of Medicine, Charles University and General University Hospital, 12800 Prague, Czech Republic; vrablikm@seznam.cz; 4 Department of Preventive Cardiology and Lipidology, Medical University of Lodz and Polish Mother’s Memorial Hospital Research Institute, 93-338 Lodz, Poland; maciejbanach@aol.co.uk; 5 Center for Endocrinology, Diabetes and Preventive Medicine, Faculty of Medicine and University Hospital Cologne, University of Cologne, 50923 Cologne, Germany

**Keywords:** lipoprotein (a), pelacarsen, olpasiran, zerlasiran, lepodisiran, muvalaplin

## Abstract

Lipoprotein (a) [Lp(a)] has been recognized as an independent, inherited, causal risk factor for atherosclerotic cardiovascular disease (ASCVD) and aortic valve stenosis, thus representing a major target of residual CV risk. Currently, no drug has been officially approved for lowering Lp(a) levels, and in clinical practice, Lp(a) is mainly used to (re)define CV risk, particularly in individuals at borderline CV risk and people with a family history of premature coronary heart disease, according to various guidelines. Specific Lp(a)-targeted antisense oligonucleotides (ASOs) and small interfering RNA (siRNA) agents have been developed to produce substantial Lp(a) reductions via the inhibition of apo(a) synthesis in the liver. These drugs are conjugated to *N*-acetylgalactosamine (GalNAc) to ensure their binding to asialoglycoproteins, which are specifically expressed on the surface of the hepatocytes. Such drugs include pelacarsen (an injectable ASO) and olpasiran, zerlasiran, and lepodisiran (injectable siRNA agents). Muvalaplin represents another therapeutic option to lower Lp(a) levels, since it is an oral selective small molecule inhibitor of Lp(a) formation, thus potentially exerting certain advantages in terms of its clinical use. The present narrative review summarizes the available clinical data on the efficacy and safety of these investigational Lp(a)-lowering therapies, as reported in phase 1 and 2 trials. The effects of these drugs on other [aside from Lp(a)] lipid parameters are also discussed. The phase 3 CV trial outcomes are ongoing for some of these agents (i.e., pelacarsen, olpasiran, and lepodisiran) and are briefly mentioned. Overall, there is an urgent need for evidence-based guidelines on Lp(a) reduction in daily clinical practice, following the results of the phase 3 CV trials, as well as for establishing the ideal Lp(a) quantification method (i.e., using an apo(a) isoform-independent assay with appropriate calibrators, reporting the Lp(a) level in molar units).

## 1. Introduction

Lipoprotein (a) [Lp(a)] represents one of the major targets for tackling residual cardiovascular (CV) risk [1,2], and it has been recognized as an independent risk factor for atherosclerotic CV disease (ASCVD) and aortic valve stenosis [3]. In daily clinical practice, Lp(a) should be used to (re)define CV risk, especially in people with a family history of premature coronary heart disease and in individuals at borderline or intermediate CV risk [4,5]. In this context, the American Heart Association (AHA) and the International Atherosclerosis Society (IAS) recommend the use of Lp(a) as a risk-enhancing factor by adjusting the 10-year risk estimate based on the following formula: predicted 10-year risk × [1.11^(patient’s Lp(a) level in nmol/L/50)^] among individuals at borderline (5–7.4%) or intermediate (7.5–19.9%) 10-year predicted risk for ASCVD [6]. The European Atherosclerosis Society (EAS) advocates for using a risk calculator for risk reclassification and setting treatment goals (https://www.lpaclinicalguidance.com/, accessed 12 May 2025). Therefore, Lp(a) can improve risk stratification and contribute to better prevention strategies. For example, an elevated Lp(a) level was previously shown to reclassify predicted ASCVD risk into a higher category in up to 18% of patients referred for familial hypercholesterolemia (FH) genotyping (n = 1504) [7]. Overall, and despite some earlier controversies surrounding the use of Lp(a) in risk prediction, several international scientific societies (e.g., EAS, AHA, IAS, and the Canadian Cardiovascular Society) strongly support the use of Lp(a) to more accurately predict an individual’s risk [8].

Another clinical use of Lp(a) refers to the correction of calculated low-density lipoprotein cholesterol (LDL-C) levels for Lp(a), a method that can more accurately estimate the “true” LDL-C levels in extremely high- and high-risk patients [9,10]. In this context, alirocumab-induced decreases in Lp(a) and corrected LDL-C levels independently predicted a lower risk of major adverse CV events, after adjusting for baseline lipoprotein levels and demographic and clinical characteristics in a pre-specified analysis of the Evaluation of Cardiovascular Outcomes After an Acute Coronary Syndrome During Treatment with Alirocumab (ODYSSEY) trial [11]. However, in light of the most recent results that do not support LDL-C correction in relation to CVD risk prediction, this issue still needs to be further elucidated [9].

According to current guidelines, Lp(a) levels should be measured at least once in each individual during their adult life [6,11,12,13,14]. However, in women, Lp(a) levels undergo fluctuations from menarche to postmenopause, with the levels being generally stabilized during reproductive years but notably increased during pregnancy and after menopause [15]. Based on these findings, it has been suggested to reevaluate Lp(a) levels in women in the postmenopausal period. Furthermore, according to the most recent data on Lp(a) visit-to-visit variability, which suggest that as many as 30% of patients at moderate risk can have the second measurement qualify them for the high group category (i.e., >50 mg/dL), the recommendations of the Polish Cardiac Society (PCS) and the Polish Lipid Association (PoLA) recommend more than one Lp(a) measurement for consideration in people with Lp(a) concentrations in the gray zone (i.e., 30–50 mg/dL (75–125 nmol/L)), with cut-off values for risk groups or with risk factors or diseases that may significantly affect Lp(a) levels [16]. They also recommended that remeasurement should be performed using a test whose results are expressed in nmol/L in order to better stratify the risk [16].

There are also certain challenges in relation to the use of Lp(a) in daily clinical practice, including differences in Lp(a) quantification methods, calibrators, and expression units [6,15]. For example, there is an urgent need to establish a globally accepted, high-quality, standardized assay for Lp(a) measurement, ideally expressed in nmol/L [17]. Furthermore, there are slightly different cut-offs for “elevated” Lp(a) in international guidelines, namely ≥50 mg/dL (or ≥125 nmol/L) in the AHA and IAS guidelines [6] and ≥50 mg/dL (or ≥100 nmol/L) in the Canadian Cardiovascular Society (CCS) guidelines [18], whereas the National Lipid Association (NLA) [19,20], the Polish guidelines [16] and the EAS [3] consider Lp(a) levels of <30 mg/dL (or <75 nmol/L) to be normal, 30–50 mg/dL (or 75–125 nmol/L) to be borderline, and >50 mg/dL (or >125 nmol/L) to be high. It is worth mentioning that more and more existing recommendations suggest Lp(a) measuring in individuals <18 years of age in specific circumstances (e.g., personal history of ischemic stroke or family history of premature ASCVD in the absence of other identifiable risk factors), including the updated NLA [19] and the Polish guidelines [16], highlighting that Lp(a) determination should be considered as early as possible, even in people <18 years, for risk assessment, cascade screening, monitoring, and lifestyle modification [21]. Furthermore, cascade testing for high Lp(a) levels is recommended in the settings of FH, family history of elevated Lp(a), personal or family history of premature ASCVD, and personal history of calcific valvular aortic stenosis and recurrent or progressive ASCVD, despite optimal lipid-lowering therapy [3,6,20]. Table 1 summarizes the above recommendations for Lp(a) measurement and use in clinical practice from different scientific societies.

Taking into consideration all of the above, scientific research has been focused on developing methods to lower Lp(a) levels in clinical practice. In this context, the most effective clinically available method for reducing Lp(a) levels to date is lipoprotein apheresis, which is mainly performed weekly or bi-weekly and leads to an acute decrease in Lp(a) of 70–85% following a single 3–4 h session, but the mean interval Lp(a) reduction remains only in the range of 25–40% [6,22]. Of note, albeit lacking prospective, randomized, CV endpoint trials showing a clinical benefit, there are some smaller studies which have reported an apheresis-related reduction in CV events in patients with high Lp(a) levels [23]. Statins unfortunately do not exert a clinically meaningful decrease in Lp(a) concentrations. Some authors even suggested that a minimal increase in Lp(a) can be seen with statin treatment (with pitavastatin affecting Lp(a) to a lesser extent) [24,25]. Ezetimibe has neutral or minimal Lp(a) reducing effects, whereas bempedoic acid seems not to affect Lp(a) levels [24,26,27]. Regarding commercially available proprotein convertase subtilisin kexin type 9 (PCSK9) inhibitors, both monoclonal antibodies (evolocumab and alirocumab) and inclisiran significantly lower Lp(a) levels by up to 20–30% [26,28,29]. However, it needs to be emphasized that the effects of statins, niacin, and especially PCSK9 inhibitors on Lp(a) levels depend on apo(a) isoforms, which are still not measured in the clinical practice and would allow predicting the response of a PCSK9-targeted approach and other therapies [21]. Nevertheless, they were correctly not approved for this indication (with targeted CVOT trials lacking and almost impossible to design since PCSK9 inhibitors massively decrease LDL-C levels). It should also be mentioned that this lack of or limited effect of the above-mentioned lipid-lowering therapies on Lp(a) levels relates to the mechanism of action of these drugs (targeting LDL-C receptors and cholesterol synthesis) versus the unique structure and synthesis of Lp(a). Of note, drug-induced Lp(a) reduction is related to the apo(a) isoforms; the presence of each additional kringle domain is associated with a further 3% Lp(a) reduction. In other words, the larger the size of the dominant apo(a) isoform, the greater the relative Lp(a) reduction [16,30]. Dietary intervention and nutraceuticals show only a modest, clinically irrelevant reducing effect on Lp(a) [31,32,33]. Interestingly, recent clinical studies with a novel inhibitor of the cholesteryl-ester transfer protein (CETP) obicetrapib have documented up to 57% lowering of Lp(a) levels with the drug [34]. The results of the BROOKLYN trial that included heterozygous FH patients, which were released at the AHA Congress 2024 in Chicago, showed that obicetrapib reduced Lp(a) levels by 54.3% (placebo-adjusted) and 38% among patients which had at least 50% Lp(a) reduction with this drug [35].

Importantly, it has been suggested that to attain a clinically meaningful reduction in ASCVD risk, at least an 8% decrease in Lp(a) levels must be achieved [36]. Thus, effective treatments are needed. Novel therapeutical approaches to lower Lp(a) levels have emerged, such as using antisense oligonucleotides (ASOs) and small interfering RNA (siRNA) agents targeting the synthesis of apo(a), which have the potential to decrease circulating Lp(a) levels by more than 80% [22]. These specific ASO- and siRNA-based therapies have enabled substantial Lp(a) reductions by decreasing apo(a) synthesis in the liver [37]. Furthermore, and in order to achieve specific delivery of the drugs to the liver, ASOs and siRNAs are conjugated to *N*-acetylgalactosamine (GalNAc). The formed conjugates target asialoglycoprotein receptors (ASGPRs) specifically expressed on the surface of the hepatocytes, thus ensuring the concentration of siRNAs or ASOs in the liver [37,38]. GalNAc is then rapidly cleaved, thus allowing the RNA interference therapy to degrade the mRNAs encoding apo(a). A more in-depth explanation of how these developing therapeutic agents work at the molecular level is included in previous papers [39,40].

The present narrative review summarizes the available clinical data on the efficacy and safety of investigational therapies, having as their primary target the lowering of Lp(a) levels. We focus on injectable ASOs (i.e., pelacarsen) and siRNAs (i.e., olpasiran, zerlasiran and lepodisiran) as well as on muvalaplin (an oral small molecule inhibitor of Lp(a) formation). Gene editing to lower Lp(a) levels is also being investigated [41], but it is not discussed here, since it is still at a pre-clinical level.

## 2. Literature Search Strategy

This is a narrative review. We searched PubMed using the following list of keywords and MeSH terms: “lipoprotein a”, or, “Lp(a)”, or, “Lp(a) lowering”, or, “Lp(a)-reducing drugs”, or, “pelacarsen”, or, “olpasiran”, or, “zerlasiran”, or, “lepodisiran”, or, “muvalaplin”. The search was restricted to articles published in English, irrespective of publication date. Data extraction from the identified studies was mainly focused on the study design details and results. As in all narrative reviews, selection bias cannot be excluded.

## 3. Pelacarsen (TQJ230)

Pelacarsen is a GalNAc-conjugated ASO (formerly known as IONIS-APO(a)-LRx and AKCEA-APO(a)-LRx) that binds to the mRNA transcript of the LPA gene, destroys it with the help of RNAases, and thus inhibits apo(a) synthesis in the liver [42]. Interestingly, pelacarsen was first developed without the GalNAc conjugation, leading to dose-dependent mean Lp(a) reductions ranging from 39.6 to 77.8% in a phase 1 trial involving healthy participants with baseline Lp(a) levels ≥25 nmol/L (100 mg/dL) [43]. Following the GalNAc conjugation of pelacarsen, the drug potency increased by a factor of 15–30, thus leading to Lp(a) decreases ranging from 66 to 92% at day 30 after multidose pelacarsen subcutaneous administration among healthy volunteers with a baseline Lp(a) level ≥75 nmol/L in a phase 2a trial [44]. In the latter study, pelacarsen also significantly reduced the mean levels of LDL-C (ranging from −13.0% to −23.9%), apoB (ranging from −11.3% to −18.5%), and oxidized phospholipids associated with apoB (ranging from −35.2% to −42.5%) and with apo(a) (ranging from −26.6% to −36.7%) [44].

In a double-blind, randomized, placebo-controlled, dose-ranging phase 2 trial (NCT03070782), 286 patients with established ASCVD and elevated baseline Lp(a) levels ≥150 nmol/L (60 mg/dL) were randomized to receive either a placebo or pelacarsen at a dosage of 20, 40, or 60 mg every 4 weeks, 20 mg every 2 weeks, or 20 mg every week [45]. Dose-dependent mean Lp(a) decreases were observed following 6 months of pelacarsen administration: −35% in the 20 mg every 4 weeks group, −56% in the 40 mg every 4 weeks group, −58% in the 20 mg every 2 weeks group, −72% in the 60 mg every 4 weeks group, and −80% in the 20 mg every week group, compared with a 6% reduction in the placebo group [45]. Of note, Lp(a) levels returned to their baseline values within 16 weeks after the last drug dose. The mean levels of oxidized phospholipids for apoB and apo(a) were also decreased in all pelacarsen groups, ranging from −37% to −88% for apoB and from −28% to −70% for apo(a) [45]. Pelacarsen was safe and well tolerated, with mild injection-site reactions being the most frequent adverse event [45].

The phase 3, double-blind, randomized, placebo-controlled Lp(a) HORIZON(a) trial (NCT04023552) is ongoing and evaluates the effects of pelacarsen on the risk of expanded major adverse CV events (MACEs) [defined as CVD death, non-fatal myocardial infarction, non-fatal stroke, and urgent coronary revascularization requiring hospitalization] among patients with established ASCVD and Lp(a) levels either ≥70 mg/dL or ≥90 mg/dL [46]. The study duration is approximately 4 years, and pelacarsen is administered at a dose of 80 mg every month, representing the equivalent of the 20-mg weekly dose in the abovementioned phase 2 study (NCT03070782) that led to a mean 80% reduction in Lp(a) levels [45]. Of note, the trial design of the Lp(a) HORIZON(a) trial has just been published [47]. The topline results of this phase 3 trial with pelacarsen are expected in 2026 (i.e., later than initially expected) due to the small number of events observed (it is an event-driven study), and if positive, then it will give the green light for the approval of first-in-class drug therapy specifically targeting elevated Lp(a) levels, an inherited, independent, and causal risk factor for ASCVD.

## 4. Olpasiran (AMG890)

Olpasiran is a first-in-class siRNA agent which is also GalNAc conjugated and prevents apo(a) synthesis via the degradation of the apo(a)-encoding mRNA. The first phase 1 study with olpasiran (NCT03626662) was a double-blind, randomized, placebo-controlled, single dose escalation trial involving 79 participants with elevated baseline Lp(a) [48]. Olpasiran was reported to dose-dependently decrease Lp(a) levels at a rate ranging from 71 to 97%, with maximum reductions occurring between days 43 and 71 after the single dose administration [48]. Thereafter, Lp(a) concentrations gradually increased, although the levels remained below those achieved by the placebo at day 225. Of note, the lowered Lp(a) concentrations lasted for several months with all doses (i.e., 9, 30, 75, and 225 mg) except the 3-mg single dose [48]. Olpasiran was well tolerated, with no serious adverse events reported. The drug reached its mean maximum plasma concentration within 7.5 h after administration, with the majority of the drug being cleared from circulation in 2–3 days [48].

The phase 2 randomized, double-blind, placebo-controlled Olpasiran Trials of Cardiovascular Events and Lipoprotein(a) Reduction-Dose Finding Study (OCEAN(a)-DOSE trial; NCT04270760) enrolled 281 patients with established ASCVD and baseline Lp(a) levels >150 nmol/L receiving either olpasiran (10 mg every 12 weeks, 75 mg every 12 weeks, 225 mg every 12 weeks, or 225 mg every 24 weeks) or a placebo [49]. Mean Lp(a) levels were increased by 3.6% in the placebo group at 36 weeks, whereas they were dose-dependently reduced in the olpasiran groups, with placebo-adjusted mean percent changes of −70.5% (95%CI, from −75.1 to −65.9) in the 10-mg group, −97.4% (95%CI, from −102.0 to −92.8) in the 75-mg dose group, −101.1% (95%CI, from −105.8 to −96.5) in the 225-mg dose group given every 12 weeks, and −100.5% (95%CI, from −105.2 to −95.8) in the 225-mg dose administered every 24 weeks [49]. The corresponding values for the placebo-adjusted mean percent change in the Lp(a) level at 48 weeks were −68.5% (95%CI, from −74.3 to −62.7), −96.1% (95%CI, from −101.9 to −90.3), −100.9% (95%CI, from −106.7 to −95.0), and −85.9% (95%CI, from −91.8 to −80.1). Regarding the impact of olpasiran on other lipids, the placebo-adjusted mean percent changes in the LDL-C and apoB levels at 36 weeks ranged from −22.6% to −24.8% and −16.7% to −18.9% across the olpasiran groups, respectively [49]. Adverse events were similar across all trial groups, and olpasiran was well tolerated, with the most common side effect being mild injection site reactions (primarily pain) [49]. A recently published analysis from the OCEAN(a)-DOSE trial reported that olpasiran did not significantly affect high-sensitivity C-reactive protein (hsCRP) or interleukin-6 levels compared with a placebo at weeks 36 or 48 [50]. In contrast, olpasiran treatment led to a significant and sustained reduction in OxPL-apoB levels [50].

The results of the phase 2 OCEAN(a)-DOSE trial supported the safety and Lp(a)-lowering efficacy of olpasiran in ASCVD patients, suggesting that a more frequent dosing schedule (i.e., 12 weeks) could be optimal, since the longer dosing interval (i.e., every 24 weeks) exhibited a smaller effect in reducing Lp(a) production. Olpasiran is currently being evaluated in the double-blind, randomized, placebo-controlled phase 3 OCEAN(a)-Outcomes trial (NCT05581303) enrolling ASCVD patients with baseline Lp(a) levels ≥200 nmol/L [51]. The results of the Lp(a) HORIZON(a) and OCEAN(a)-Outcomes trial, when available, will further contribute to our understanding of how much Lp(a) levels should be reduced in order to have a meaningful CVD risk reduction [51].

## 5. Zerlasiran (SLN360)

Zerlasiran, a 19-mer siRNA, is also being covalently linked to the tri-antennary GalNac moiety as well as being investigated in terms of Lp(a)-lowering efficacy and safety. In a single dose-ascending study (NCT04606602) involving 32 adults with Lp(a) levels >150 nmol/L, zerlasiran was reported to dose-dependently lower Lp(a) levels; the maximal median percentage changes were −98% (IQR, from −98% to −97%) in the 600-mg dose group, −96% (IQR, from −98% to −89%) in the 300-mg group, −86% (IQR, from −92% to −82%) in the 100-mg group, −46% (IQR, from −64% to −40%) in the 30-mg group, and −10% (IQR, from −16% to 1%) in the placebo group [52]. Notably, the lowest Lp(a) levels achieved were observed between 30 and 60 days after administration in all treatment groups. Furthermore, the duration of Lp(a) reduction was also dose-dependent, being persistent for at least 150 days after drug administration; the median Lp(a) levels were >70% and >80% below baseline at 150 days in the 300-mg and 600-mg groups, respectively [52]. Apart from Lp(a), apoB and oxidized LDL were also lowered with zerlasiran; the maximum apoB reduction (by 24%) was measured 30 days after administration of the 600-mg dose, whereas the maximum apoB decrease (by 19%) after the 300-mg dose was measured at day 14 [52]. The maximum reductions in the mean oxidized LDL levels were 20% in the zerlasiran 600-mg dose group and 11% in the 300-mg dose group, with similar decreases being sustained for up to 150 days in the 600-mg group [52]. Furthermore, zerlasiran led to a dose-dependent decrease in both the total and LDL-C levels, reaching a maximum reduction of 18% for the total and 26% for LDL-C in the 600-mg dose group [52]. In contrast, zerlasiran did not affect the triglyceride or HDL-C levels. In this phase 1 study, zerlasiran was well tolerated; low-grade injection site reactions were observed that were self-limiting and did not cause participant withdrawal [52]. Of note, dose-dependent increases in the CRP levels were observed; the maximum increase was recorded in the 600-mg dose group, from a median of 1.0 mg/L at baseline to 3.6 mg/L at day 7, which then rapidly decreased. Similarly, neutrophils were raised during the first day, returning to normal values by day 7 [52].

As prespecified in the protocol, this phase 1 trial (NCT04606602) subsequently followed up with 13 out of the 32 healthy participants for an extended period of time (i.e., 365 days), as well as an additional 36 enrolled adult patients with ASCVD and baseline Lp(a) levels ≥150 nmol/L (60 mg/dL) that received two doses of a placebo, 200 mg of zerlasiran at a 4-week interval, or 300 mg or 450 mg at an 8-week interval [53]. After single doses, at day 365, the median Lp(a) levels increased by 14% (IQR, 13–15%) in the placebo group, whereas in the zerlasiran groups, similar median Lp(a) reductions were observed (i.e., −30% (IQR, from −51% to −18%) in the 300-mg zerlasiran group and −29% (IQR, from −39% to −7%) in the 600-mg group) [53]. After two doses, the median Lp(a) concentration was increased by 7% (IQR, from −4% to 21%) in the placebo group, whereas dose-dependent Lp(a) decreases were observed in the zerlasiran groups. The median Lp(a) changes were −97% (IQR, from −98% to −95%) in the 200-mg zerlasiran group and −98% (IQR, from −99% to −97%), and −99% (IQR, from −99% to −98%) in the 300-mg and 450-mg groups, respectively [53]. At day 201, the median changes in the Lp(a) values were +0.3% (IQR, from −2% to 21%), −60% (IQR, from −71% to −40%), −90% (IQR, from −91% to −74%), and −89% (IQR, from −91% to −76%) in the placebo, 200 mg, 300 mg, and 450-mg groups, respectively [53].

The effects of zerlasiran on apoB and LDL-C were also measured after multiple doses [53]. The maximal median changes in the LDL-C levels were +17% (IQR, from −2% to 29%) for the placebo group (observed at day 150), −35% (IQR, from −45% to −26%) for the 200-mg dose group (observed at day 60), −47% (IQR, from −64% to −12%) for the 300-mg group (observed at day 30), and −28% (IQR, from −38% to −26%) for the 450-mg group (observed at day 90) [47]. Similarly, the maximal median changes in the apoB levels were +12% (IQR, from 2% to 17%) for the placebo group (at day 150), −26% (IQR, from −35% to −8%) for the 200-mg group (at day 43), −28% (IQR, from −37% to −21%) for the 300-mg group (at day 90), and −23% (IQR, from −34% to −22%) for the 450-mg group (at day 90) [53]. Oxidized LDL-C levels were dose-dependently reduced, with a mean maximal change of −26% observed in the 450-mg group at day 30 [53].

The drug was well tolerated, and no serious adverse events occurred [53]. Of note, the CRP levels were dose-dependently raised during the first 24 h [median (IQR) increases were 1.4 (0.7–1.9), 2.2 (1.2–5.1), and 4.1 (2.0–19.3) mg/L for the 200 mg, 300 mg, and 450-mg groups, respectively, whereas there was a median decrease (IQR) of −0.1 (from −0.2 to 0.01) mg/L in the placebo group] but returned to normal values by day 7 and remained the same thereafter [53].

Overall, zerlasiran was well tolerated and safe in this small group of healthy adults and patients with stable ASCVD following the administration of either single doses (up to 600 mg) or two doses of 200, 300, or 450 mg [53]. Two doses of zerlasiran were shown to reduce Lp(a) levels by up to 99% (98% in the 300-mg group and 99% in the 450-mg group) at day 60, with the corresponding changes at day 201 being −90% and −89%, respectively [53]. These findings strongly supported the safety and efficacy of zerlasiran and thus led to the conduction of a larger phase 2 trial (NCT05537571), specifically the ALPACAR-360 trial, the results of which were recently published [54]. In this placebo-controlled, randomized, double-blind clinical trial, 178 patients with stable ASCVD (defined as having a prior myocardial infarction, stroke, coronary artery disease, peripheral artery disease, or computed tomography-detected coronary calcium) and a baseline Lp(a) level of ≥125 nmol/L were randomized to receive either a placebo (three doses every 16 weeks or two doses every 24 weeks) or zerlasiran (two doses of 450 mg every 24 weeks, three doses of 300 mg every 16 weeks, or two doses of 300 mg every 24 weeks) and followed up for 60 weeks [54]. Interestingly, the primary outcome of this study was the time-averaged percent change in Lp(a) levels from baseline to 36 weeks (and not Lp(a) maximal reductions), thus more accurately reporting the effects of treatment over time. In brief, the least-squares mean, placebo-adjusted, time-averaged percent change in Lp(a) levels (from baseline to week 36) was greater than 80% in all groups: −85.6% (95%CI, from −90.9% to −80.3%) for the 450-mg group (given every 24 weeks), −82.8% (95%CI, from −88.2% to −77.4%) for the 300-mg group (administered every 16 weeks), and −81.3% (95%CI, from −86.7% to −76.0%) for the 300-mg group (given every 24 weeks) [54]. Furthermore, the corresponding median (IQR) percent reductions in Lp(a) levels at week 36 were −94.5% (from −97.3% to −84.2%), −96.4% (from −97.7% to −92.3%), and −90.0% (from −93.7% to −81.3%), respectively. In this phase 2 trial, zerlasiran was safe and well tolerated; the most frequent side effect was injection site reactions with mild pain [54].

These findings strongly support the conduction of a larger phase 3 trial of zerlasiran, which is assumed to be already in planning.

## 6. Lepodisiran (LY3819469)

Lepodisiran is another GalNAc-conjugated siRNA that was reported to produce durable reductions in Lp(a) levels in a single ascending-dose study (NCT04914546) with a maximum follow-up of 336 days (48 weeks) after a administration of a single dose [55]. In brief, this phase 1 study involved 48 adults without ASCVD and with baseline Lp(a) levels ≥30 mg/dL (75 nmol/L) from the US and Singapore that were randomized to receive either a placebo or a single dose of lepodisiran (4 mg, 12 mg, 32 mg, 96 mg, 304 mg, or 608 mg) administered subcutaneously [55]. The plasma lepodisiran concentrations reached peak levels within 10.5 h and became undetectable by 48 h. Lp(a) levels were dose-dependently reduced, with the maximal median change being −97% (IQR, from −98% to −96%) in the 608-mg dose group, −96% (IQR, from −98% to −95%) in the 304-mg group, −90% (IQR, from −94% to −85%) in the 96-mg group, −76% (IQR, from −76% to −75%) in the 32-mg group, −59% (IQR, from −66% to −53%) in the 12-mg group, −41% (IQR, from −47% to −20%) in the 4-mg group, and −5% (IQR, from −16% to 11%) in the placebo group [55]. Interestingly, these maximal Lp(a) changes were observed at day 29 for the 608-mg dose group, at day 43 for the 96- and 304-mg groups, at day 57 for the 32-mg group, at day 43 for the 12-mg group, and at day 85 for the 4-mg group. Furthermore, the percentage changes in Lp(a) concentrations lasted longer with the highest doses of lepodisiran; at day 337, the median change in Lp(a) concentration was −94% (IQR, from −94% to −85%) in the 608-mg lepodisiran group, demonstrating that the administration of a single high dose of lepodisiran can lead to durable Lp(a)-lowering effect to the range of >90% [55]. Based on these findings, lepodisiran (especially at the highest doses) may be administrated only once or twice yearly and still be able to achieve a high degree of Lp(a)-lowering efficacy. In this phase 1 study, lepodisiran was well tolerated, with adverse events being uncommon and generally similar across lepodisiran dose groups and the placebo group [55].

A phase 2, randomized, double-blind, placebo-controlled study (NCT05565742) investigating the efficacy and safety of lepodisiran among 320 adults with elevated baseline Lp(a) levels (≥175 nmol/L), followed up for up to 20 months was completed, and the results were quite recently published [56]. Participants were randomized to receive lepodisiran at a 1:2:2:2:2 ratio at a dose of 16, 96, or 400 mg at baseline and at day 180, lepodisiran at a dose of 400 mg at baseline and a placebo at day 180, or a placebo at baseline and at day 180 [56]. The placebo-adjusted, time-averaged percent change in Lp(a) levels from day 60 to day 180 was −40.8% (95%CI, from −55.8 to −20.6), −75.2% (95%CI, from −80.4 to −68.5), and −93.9% (95%CI, from −95.1 to −92.5) in the 16-mg and 96-mg lepodosiran groups and in the pooled 400-mg groups, respectively [56]. No serious safety issues were recorded, as mainly dose-dependent, mild injection site reactions occurred (up to 12% in the highest lepodisiran dose group) [56]. A phase 3 CV outcomes study is ongoing, entitled “A Study to Investigate the Effect of Lepodisiran on the Reduction of Major Adverse Cardiovascular Events in Adults with Elevated Lipoprotein(a)-ACCLAIM-Lp(a)” (NCT06292013), which will involve approximately 12,500 patients with ASCVD (aged >18 years with an event or revascularization) or at risk high risk of ASCVD (aged >55 years with atherosclerotic disease and without a clinical event, revascularization, FH, or presence of multiple risk factors) and with a baseline Lp(a) level ≥175 nmol/L [57]. Of note, the ACCLAIM-Lp(a) trial is the first clinical study with Lp(a)-targeted therapy that has a primary prevention arm.

## 7. Muvalaplin (LY3473329)

Lp(a) can be effectively lowered by the above-mentioned ASO- and siRNA-based therapies. However, these drugs are injectable, and thus certain limitations, including injection site reactions (although mild), may hinder their use [58]. In this context, an oral Lp(a)-reducing therapeutical agent (i.e., muvalaplin) has been developed [58]. Muvalaplin is the first oral agent that specifically lowers circulating Lp(a) levels by blocking the interaction of apo(a) KIV domains 7 and 8 with the lysine in apoB-100, thus inhibiting Lp(a) formation in the liver [58]. This drug’s efficacy in lowering Lp(a) levels was first shown in animal studies involving transgenic mice and cynomolgus monkeys [59].

In a single-center, randomized, double-blind phase 1 trial (NCT04472676), among 114 healthy participants, 55 participants (with any Lp(a) level) received a single ascending dose of muvalaplin (ranging from 1 to 800 mg) or a placebo, whereas the remaining 59 participants (with Lp(a) ≥30 mg/dL) received multiple ascending doses of muvalaplin (ranging from 30 to 800 mg daily) or a placebo for 14 days [60]. The mean (±SD) age of the participants was 29 (±20) years in the single dose group (64% females) and 32 (±15) years in the multiple dose group (58% females); the corresponding levels of the median baseline Lp(a) were 10 mg/dL and 58 mg/dL, respectively [60]. In the single dose group, muvalaplin reached its maximum plasma concentration 2–5 h after administration, followed by a multiphasic decline, with an elimination half-life of 12–67 h [60]. In the multiple dose group, the maximum plasma muvalaplin concentration occurred between 2 and 6 h after administration on day 1 and between 2 and 4 h after dosing on day 14, with the elimination half-life ranging from 70 to 414 h [60]. Dose-dependent reductions in Lp(a) levels were observed as early as day 2, with the placebo-controlled Lp(a) decrease ranging from 63 to 65% at doses of 100 mg or more at day 14 [60]. This effect led to achieving Lp(a) levels <50 mg/dL in 93% of the participants in the multiple dose group, with similar effects at daily doses of 100 mg or more. No clinically significant safety or tolerability concerns were reported. In this context, observed adverse events (mainly headache, fatigue, vomiting, back pain, nausea, and diarrhea) were few in number and mild [60]. Similarly, no hematological or liver biochemical side effects were observed; muvalaplin exerted only a minimal and not clinically significant impact on plasminogen activity (i.e., up to 14% reduction) independent of the dosage [60].

Interestingly, it has been suggested that current commercially available Lp(a) assays may be insufficient to accurately determine the Lp(a)-lowering effect of muvalaplin, since they measure the total apo(a) level and not intact Lp(a) [61]. In contrast, an apo(a) isoform-independent assay that measures only Lp(a) particles is more appropriate for estimating the Lp(a)-reducing efficacy of muvalaplin [21,61]. In this context, further discussion on the different Lp(a) assays and the relevant recommendations from international scientific societies are included in the Section 8 below. A recently published phase 2 randomized, placebo-controlled, double-blind trial (KRAKEN trial-NCT05563246), including 233 patients with ASCVD, FH, or diabetes and having baseline Lp(a) levels of ≥175 nmol/L, used both an assay to measure intact Lp(a) levels and a traditional apo(a)-based assay to measure the molar concentration [62]. Patients (median age: 66 years; 33% females) received either a placebo (n = 67) or muvalaplin at doses of 10 mg/day (n = 34), 60 mg/day (n = 64), or 240 mg/day (n = 67) for 12 weeks. Muvalaplin led to placebo-adjusted Lp(a) reductions of 47.6% (95%CI, 35.1–57.7%), 81.7% (95%CI, 78.1–84.6%) and 85.8% (95%CI, 83.1–88.0%) for the 10-, 60-, and 240-mg/day dosages, respectively, using an intact Lp(a) assay [62]. The corresponding reductions using an apo(a)-based assay were 40.4% (95%CI, 28.3–50.5%), 70.0% (95%CI, 65.0–74.2%), and 68.9% (95%CI, 63.8–73.3%). Of note, dose-dependent decreases in apoB levels also occurred [8.9% (95%CI, from −2.2% to 18.8%), 13.1% (95%CI, 4.4–20.9%), and 16.1% (95%CI, 7.8–23.7%) in the 10-, 60-, and 240-mg group, respectively], whereas no change in the high-sensitivity CRP levels was observed. Furthermore, no safety or tolerability concerns were observed at any dose, with adverse events being similar in both the muvalaplin and placebo groups (5.9, 14.3, and 14.7% in the 10-, 60-, and 240-mg muvalaplin groups, respectively, versus 14.9% in the placebo group) [62].

Overall, muvalaplin, when being orally administered, seems to have certain advantages, such as potential patient acceptability, a hopefully lower price, and convenience, which may improve patient adherence [58]. We should also keep in mind that the currently used apo(a)-based immunoturbidometric Lp(a) assays may underestimate the Lp(a)-reducing effect of muvalaplin, since they measure apo(a) bound to both muvalaplin and apoB [61,63]. Furthermore, a large phase 3 clinical trial evaluating the impact of muvalaplin on CV outcomes is currently planned.

Table 2 summarizes the effects of investigational Lp(a)-lowering agents on Lp(a) and other lipid parameters in phase 1 and 2 clinical trials.

## 8. Discussion

As discussed above, certain Lp(a)-lowering therapies have been developed and are currently being investigated in clinical trials in order to establish their use, if any, in daily clinical practice. The results of the available clinical studies with such therapies should be interpreted with caution, since there are several limitations, including the relatively small number of participants and the short follow-up period, which is not sufficient to assess the long-term effects of therapy, as well as the lack of data on “hard” clinical outcomes (e.g., reduction of CV events), as most research is focused on examining biomarkers. In this context, the results of the phase 3 CV outcome trials (e.g., Lp(a) HORIZON(a), OCEAN(a)-Outcomes, and ACCLAIM-Lp(a)) are awaited with much interest. However, we also need to consider that the results of these CV trials may prove insufficient to adopt new therapeutic guidelines according to their findings, and thus additional studies may be needed.

Another consideration refers to the relatively high treatment cost of “biological therapies”, together with difficulties in relation to the availability and implementation of such treatments in daily clinical practice that the health systems of many countries have to face. However, the existing lipid-lowering therapies that have been shown to reduce Lp(a) levels are either also expensive and difficult to implement in daily practice (i.e., lipoprotein apheresis) or have not been approved for Lp(a) lowering (e.g., PCSK9 inhibitors). Needless to say, these drugs can decrease Lp(a) levels to a much lesser extent compared with the specific ASO- and siRNA-based therapies. Notably, cost-effectiveness studies should be performed to support the clinical use of the specific Lp(a)-reducing drugs despite their high cost. The potential clinical use of orally administered muvalaplin, if approved, may represent an attractive option with certain benefits (e.g., low cost, patient acceptability, and better adherence), as discussed above.

Regarding the need for standardization of Lp(a) measurement methods, it is important to emphasize that international scientific societies (i.e., EAS, AHA, and IAS) discuss this issue. For example, the EAS recommends that “*clinical assays should use an antibody for a unique non-repetitive epitope in apo(a), recognizing each Lp(a) particle once and reporting levels as nmol/L. In practice, as raising such antibodies is difficult, most assays incorporate polyclonal antibodies which recognize different epitopes, and therefore potentially underestimate or overestimate Lp(a) levels depending on the presence of small or large isoforms, respectively. Incorporating multiple calibrators spanning a range of sizes in the assay can at least partly address this issue.*” [3]. The AHA and IAS recommend that “*Lp(a) should be measured with an isoform-insensitive assay, an assay that is traceable to the internationally accepted calibrator (World Health Organization/International Federation of Clinical Chemistry Reference Material SRM-2B) or an assay that is reported in nanomoles per liter (nmol/L)*.” [6]. Both the EAS and the AHA and IAS strongly highlight the need to improve the standardization and harmonization of Lp(a) measurement [3,6]. Over, handling the challenges of standardizing Lp(a) measurement is a crucial point, as different assays can yield different results, making it difficult to compare Lp(a) levels across studies and populations.

As mentioned in the Introduction of the present narrative review, Lp(a) synthesis is under a strong genetic influence, whereby polymorphisms of certain genes and enzymes may affect the effectiveness of therapy. Therefore, it is logical to consider whether prior genetic testing could be necessary for individualizing therapeutical options and predicting responses to treatment. No relevant data are available to date. In this context, the EAS guidelines mention that “*many aspects of the genetic regulation of Lp(a) are not fully understood. Identification of causal variants and the mechanisms by which they modulate Lp(a) concentration or enhance Lp(a) pathogenicity require further research*” [3]. Furthermore, the AHA and IAS guidelines prioritize the need for decoding the mechanisms by which genes other than *LPA* can regulate Lp(a) levels (such as *APOE* or *APOH*) [6].

As already mentioned, this is a narrative review with certain limitations, including potential biases in the selection of studies and the interpretation of findings. A systematic review and, ideally, a meta-analysis would minimize these limitations. As more clinical data are being published, these are therefore necessary. Furthermore, a critical appraisal of the quality of the clinical trials included in this narrative review should be considered (e.g., confidence intervals and heterogeneity, the risk of bias (potentially minimized with the use of the Cochrane Risk of Bias tool for randomized controlled trials), sample size, statistical power (particularly for phase 1 and 2 trials, which may be underpowered to detect clinically meaningful differences), and generalizability (considering the characteristics of the study populations and whether the findings are likely to be applicable to broader patient groups).

Finally, it is important to acknowledge that the optimal target level for Lp(a) reduction is still debated. It is not yet clearly established how much Lp(a) levels need to be lowered to achieve a significant clinical benefit, although a previous Mendelian randomization analysis showed that a 101.5 mg/dL drop in Lp(a) levels had the same impact on CHD risk as a 38.67 mg/dL reduction in the LDL-C concentration [36]. Based on the analysis of data from previous population base studies (i.e., the Copenhagen General Population Study (CGPS), the Copenhagen City Heart Study (CHS), and the Copenhagen Ischemic Heart Disease Study (CIHDS)), an Lp(a) level reduction of 50 mg/dL (105 nmol/L) in the short term (i.e., 5 years) may be needed to decrease CV risk by 20% in secondary prevention settings [64].

## 9. Conclusions

An elevated Lp(a) level has been recognized as an established, inherited, independent risk factor for ASCVD and aortic valve stenosis. Specific GalNAc-conjugated ASO- and siRNA-based therapies and targeted apo(a) synthesis have been developed, enabling substantial Lp(a) reduction. Such drugs include pelacarsen (an injectable ASO) and olpasiran, zerlasiran, and lepodisiran (injectable siRNA agents). Muvalaplin represents another therapeutic option to lower Lp(a) levels, since it is an oral small molecule inhibitor of an Lp(a) formation. The safety of and efficacy in Lp(a) lowering among these drugs have been shown in phase 1 and 2 trials, whereas phase 3 CV trials are ongoing for pelacarsen, olpasiran, and lepodisiran. The results of these CV endpoint trials are of fundamental importance, since they will not only show (hopefully) that decreasing Lp(a) levels translates to a decrease in CV events and CV death, but most importantly, they will, after decades of uncertainty, finally bring the endless saga of Lp(a) to its end, being either the potential silent killer hidden in plain site for decades or the falsely accused innocent bystander. However, the efficacy of these novel Lp(a)-lowering therapies in terms of clinical outcomes is yet to be determined. Furthermore, long-term safety data are lacking, and thus larger trials with longer durations are needed to fully assess the safety profiles of these agents.

## Figures and Tables

**Table 1:** Recommendations for Lp(a) measurement and use in clinical practice from different scientific societies.

Scientific Society	Recommendations
American Cardiology College (ACC) and American Heart Association (AHA) [6]	Lp(a) levels should be measured at least once in each individual during their adult life Cascade screening of family members of patients with elevated Lp(a) levels Use of Lp(a) as a risk-enhancing factor by adjusting the 10-year risk estimate based on the following formula: predicted 10-year risk × [1.11^(patient’s Lp(a) level in nmol/L/50)^] among individuals at borderline (5–7.4%) or intermediate (7.5–19.9%) 10-year predicted risk for ASCVD Elevated Lp(a): ≥50 mg/dL (or ≥125 nmol/L) Lp(a) should be measured with the following: ➢ An isoform-insensitive assay ➢ An assay that is traceable to the internationally accepted calibrator (World Health Organization/International Federation of Clinical Chemistry Reference Material SRM-2B) ➢ An assay that is reported in nanomoles per liter (nmol/L)
European Atherosclerosis Society (EAS) [3]	Lp(a) levels of <30 mg/dL (or <75 nmol/L) are normal, while 30–50 mg/dL (or 75–125 nmol/L) is borderline and >50 mg/dL (or >125 nmol/L) is high Lp(a) levels should be measured at least once in each individual during their adult life In youths, Lp(a) measurement is recommended when there is a history of ischemic stroke or a parent has premature ASCVD and no other identifiable risk factors Cascade testing for high Lp(a) levels is recommended in the settings of FH, family history of (extremely) high Lp(a) levels, and personal or family history of ASCVD Use an Lp(a) assay that is insensitive to the apo(a) isoform and traceable to official reference materials Measurement of Lp(a) levels should be in molar units, if available
Canadian Cardiovascular Society (CCS) [18]	Elevated Lp(a): ≥50 mg/dL (or ≥100 nmol/L) Lp(a) levels should be measured at least once in each individual during their adult life Lp(a) measurement should be considered in younger patients, particularly those who have an extremely strong family history of premature ASCVD
Polish Cardiac Society (PCS) and the Polish Lipid Association (PoLA) [16]	Lp(a) levels of <30 mg/dL (or <75 nmol/L) are normal, while 30–50 mg/dL (or 75–125 nmol/L) is borderline and >50 mg/dL (or >125 nmol/L) is high More than one Lp(a) measurement should be considered in people with Lp(a) concentrations in the gray zone (i.e., 30–50 mg/dL (75–125 nmol/L)), with cut-off values for risk groups or with risk factors or diseases that may significantly affect Lp(a) levels It is recommended that remeasurement be performed using a test whose results are expressed in nmol/L Lp(a) levels should be measured at least once in each individual during their adult life, and for women, it is recommended to remeasure Lp(a) levels after the age of 50 years Measurement of Lp(a) levels should be considered in all patients with premature ASCVD and those with a borderline risk between moderate and high for better risk stratification Measurement of Lp(a) levels can be considered in extremely high-risk patients with ASCVD, as well as in patients with FH Screening of relatives of individuals with high Lp(a) levels should be considered Lp(a) measuring in individuals <18 years of age in specific circumstances
National Lipid Association (NLA) [19,20]	Lp(a) levels of <30 mg/dL (or <75 nmol/L) are normal, while 30–50 mg/dL (or 75–125 nmol/L) is borderline and >50 mg/dL (or >125 nmol/L) is high Clinicians should use, when possible, assays that report results in nmol/L using a 5-point or similar calibrator and which are calibrated against the WHO and International Federation of Clinical Chemistry and Laboratory Medicine secondary reference material Lp(a) measuring in adults with the following: ➢ A family history of first-degree relatives with premature ASCVD (<55 years of age in men; <65 years of age in women) ➢ A family history of elevated Lp(a) levels ➢ Premature ASCVD (<55 years of age in men; <65 years of age in women), particularly in the absence of traditional risk factors ➢ Primary severe hypercholesterolemia (LDL-C >190 mg/dL) or suspected FH ➢ Calcific valvular aortic stenosis ➢ Recurrent or progressive ASCVD, despite optimal lipid-lowering therapy ➢ Intermediate or borderline 10-year ASCVD risk to improve risk stratification in primary prevention Lp(a) measuring in individuals <18 years of age in specific circumstances (e.g., cascade screening of first-degree relatives of patients with elevated Lp(a)), measurement of Lp(a) levels in youths with a history of ischemic stroke, with clinically suspected or genetically confirmed FH, or with a family history of first-degree relatives with premature ASCVD (<55 years of age in men; <65 years of age in women)
Hellenic Atherosclerosis Society [14]	Elevated Lp(a) levels: ≥50 mg/dL or ≥125 nmol/L It is preferable to measure the Lp(a) molar concentration (in nmol/L) Lp(a) levels should be measured at least once in each person’s lifetime Lp(a) assessment should be considered for the reclassification of patients having a borderline estimated 10-year ASCVD risk

**Table 2:** Effects of investigational lipoprotein (a) [Lp(a)]-lowering agents on Lp(a) and other lipid parameters in phase I-II clinical trials.

Agent	Phase 1	Phase 2	Phase 3
Pelacarsen (TQJ230)	healthy individuals [43] **Dosing**: a single subcutaneous injection of pelacarsen (50 mg, 100 mg, 200 mg, or 400 mg) or placebo (3:1) in the single-dose part of the study or six subcutaneous injections of pelacarsen (100 mg, 200 mg, or 300 mg, for a total dose exposure of 600 mg, 1200 mg, or 1800 mg) or placebo (4:1) during a 4-week period in the multi-dose part of the study. **Baseline to day 30**: single doses of pelacarsen (50–400 mg) did not decrease Lp(a) concentrations **Baseline to day 36**: dose-dependent Lp(a) reductions by 39.6(±10.3)% in the 100-mg group, 59(±19.7)% in the 200-mg group and 77.8(±9.4)% in the 300-mg group	healthy individuals (Phase 2) [44] **Dosing**: escalating-dose subcutaneous injections of pelacarsen (100 mg, 200 mg, and then 300 mg, once a week for 4 weeks each) or placebo (in a 1:1 ratio in cohort A and in a 4:1 ratio in cohort B) **Baseline to day 85/99:** Lp(a) reductions by 66.8(±20.6)% in cohort A and 71.6(±13.0%) in cohort B mean difference from placebo: −62.8 (95%CI −71.9 to −53.8) nmol/L in cohort A and −67.7 (95%CI −80.8 to −54.5) nmol/L in cohort B LDL-C reduction: mean difference from placebo: −13.0 (95%CI −20.2 to −5.7) mmol/L in cohort A and −23.9 (95%CI −34.2 to −13.5) mmol/L in cohort B apoB reduction: mean difference from placebo: −11.3 (95%CI −17.2 to −5.4) mg/dL in cohort A and −18.5 (95%CI −27.0 to −10.0) mgl/dL in cohort B oxidized phospholipids associated with apoB reduction: mean difference from placebo: −35.2 (95%CI −43.1 to −27.2) nmol/L in cohort A and −42.5 (95%CI −54.0 to −31.0) nmol/L in cohort B oxidized phospholipids associated with apo(a) reduction: mean difference from placebo: −26.6 (95%CI −40.3 to −13.0) nmol/L in cohort A and −36.7 (95%CI −56.4 to −16.9) nmol/L in cohort B healthy individuals (Phase 2a) [44] **Dosing**: a single dose of 10–120 mg pelacarsen subcutaneously in an ascending-dose design or placebo (in a 3:1 ratio; single ascending-dose phase), or multiple doses of 10 mg, 20 mg, or 40 mg pelacarsen subcutaneously in an ascending-dose design or placebo (in an 8:2 ratio) at day 1, 3, 5, 8, 15, and 22 (multiple-ascending-dose phase). **Baseline to day 30 (single dosing):** dose-dependent Lp(a) reductions by 26.2(±5.4)% in the 10-mg group, 33.2(±17.5)% in the 20-mg group, 43.5(±14.3)% in the 40-mg group, 78.6(±21.2)% in the 80-mg group and 85.3(±7.1)% in the 120-mg group mean difference from placebo: −24.8 (95%CI −67.1 to −3.1) nmol/L in the 10-mg group, −35.1 (95%CI −78.8 to −2.2) nmol/L in the 20-mg group, −48.2 (95%CI −78.4 to −10.9) nmol/L in the 40-mg group, −82.5 (95%CI −109.2 to −50.5) nmol/L in the 80-mg group and −84.5 (95%CI −112.6 to −65.2) nmol/L in the 120-mg group LDL-C reduction: mean difference from placebo: −6.2 (95%CI −34.3 to +7.8) mmol/L in the 10-mg group, −0.6 (95%CI −24.4 to +23.1) mmol/L in the 20-mg group, −7.9 (95%CI −31.6 to +20.7) mmol/L in the 40-mg group, −8.0 (95%CI −34.3 to +1.7) mmol/L in the 80-mg group and −26.7 (95%CI −55.5 to −11.3) mmol/L in the 120-mg group. apoB reduction: mean difference from placebo: −4.9 (95%CI −17.2 to +4.5) mg/dL in the 10-mg group, −1.3 (95%CI −19.3 to +13.1) mg/dL in the 20-mg group, −10.3 (95%CI −24.4 to +1.9) mg/dL in the 40-mg group, −9.2 (95%CI −26.2 to +0.0) mg/dL in the 80-mg group and −17.1 (95%CI −31.8 to −2.5) mg/dL in the 120-mg group. oxidized phospholipids associated with apoB reduction: mean difference from placebo: −13.1 (95%CI −46.4 to +13.9) nmol/L in the 10-mg group, −10.7 (95%CI −49.0 to +33.4) nmol/L in the 20-mg group, −14.3 (95%CI −47.8 to +10.7) nmol/L in the 40-mg group, −18.3 (95%CI −44.3 to +9.7) nmol/L in the 80-mg group and −18.0 (95%CI −46.4 to +9.4) nmol/L in the 120-mg group. oxidized phospholipids associated with apo(a) reduction: mean difference from placebo: −25.4 (95%CI −152.8 to +150.7) nmol/L in the 10-mg group, −28.4 (95%CI −152.0 to +66.5) nmol/L in the 20-mg group, −11.8 (95%CI −134.4 to +61.3) nmol/L in the 40-mg group, −43.9 (95%CI −119.3 to +23.1) nmol/L in the 80-mg group and −40.9 (95%CI −143.9 to −7.5) nmol/L in the 120-mg group. **Baseline to day 36 (multiple dosing)**: dose-dependent Lp(a) reductions by 65.7(±21.8)% in the 10-mg group, 80.1(±13.7)% in the 20-mg group and 92.4(±6.5)% in the 40-mg group mean difference from placebo: −59.4 (95%CI −79.1 to −33.5) nmol/L in the 10-mg group, −72.3 (95%CI −87.7 to −51.6) nmol/L in the 20-mg group and −82.4 (95%CI −99.8 to −67.7) nmol/L in the 40-mg group LDL-C reduction: mean difference from placebo: −10.6 (95%CI −24.1 to +10.1) mmol/L in the 10-mg group, −5.7 (95%CI −30.3 to +2.3) mmol/L in the 20-mg group and −13.7 (95%CI −28.1 to −4.4) mmol/L in the 40-mg group apoB reduction: mean difference from placebo: −6.8 (95%CI −14.4 to +23.6) mg/dL in the 10-mg group, −7.4 (95%CI −20.7 to +1.7) mg/dL in the 20-mg group and −13.6 (95%CI −23.1 to −1.1) mg/dL in the 40-mg group oxidized phospholipids associated with apoB reduction: mean difference from placebo: −31.9 (95%CI −54.4 to −13.6) nmol/L in the 10-mg group, −24.1 (95%CI −44.4 to −6.0) nmol/L in the 20-mg group and −39.8 (95%CI −58.0 to −23.8) nmol/L in the 40-mg group oxidized phospholipids associated with apo(a) reduction: mean difference from placebo: −28.9 (95%CI −60.3 to −6.6) nmol/L in the 10-mg group, −49.0 (95%CI −97.9 to −16.2) nmol/L in the 20-mg group and −77.9 (95%CI −106.3 to −53.2) nmol/L in the 40-mg group patients with established ASCVD [45] **Dosing:** subcutaneous administration of pelacarsen (20, 40, or 60 mg every 4 weeks; 20 mg every 2 weeks; or 20 mg every week), or placebo for 6 months. **Baseline to 6 months:** dose-dependent Lp(a) reductions: by 95.9(±94.4) nmol/L at a dose of 20 mg every 4 weeks, 116.9(±71.7) nmol/L at 40 mg every 4 weeks, 130.3(±66.1) nmol/L at 20 mg every 2 weeks, 149.5(±67.4) nmol/L at 60 mg every 4 weeks, and 187.8(±80.3) nmol/L at 20 mg every week dose-dependent mean percent reductions in Lp(a) from baseline: 35% at a dose of 20 mg every 4 weeks, 56% at 40 mg every 4 weeks, 58% at 20 mg every 2 weeks, 72% at 60 mg every 4 weeks, and 80% at 20 mg every week, as compared with 6% for the pooled placebo group oxidized phospholipids associated with apo(a) reduction: −16.8(±14.3) nmol/L at a dose of 20 mg every 4 weeks, −24.5(±20.1) nmol/L at 40 mg every 4 weeks, −25.9(±17.2) nmol/L at 20 mg every 2 weeks, −33.3(±16.8) nmol/L at 60 mg every 4 weeks, and −41.6(±16.5) nmol/L at 20 mg every week mean percent reductions in oxidized phospholipids on apo(a): 28% at a dose of 20 mg every 4 weeks, 49% at 40 mg every 4 weeks, 45% at 20 mg every 2 weeks, 63% at 60 mg every 4 weeks, and 70% at 20 mg every week, as compared with a 20% decrease in the placebo group. oxidized phospholipids associated with apoB reduction: −8.0(±10.3) nmol/L at a dose of 20 mg every 4 weeks, −11.3(±11.0) nmol/L at 40 mg every 4 weeks, −12.2(±7.9) nmol/L at 20 mg every 2 weeks, −14.9(±10.3) nmol/L at 60 mg every 4 weeks, and −20.1(±8.5) nmol/L at 20 mg every week mean percent reductions in oxidized phospholipids on apoB: 37% at a dose of 20 mg every 4 weeks, 57% at 40 mg every 4 weeks, 64% at 20 mg every 2 weeks, 79% at 60 mg every 4 weeks, and 88% at 20 mg every week, as compared with a 14% increase in the placebo group.	patients with established ASCVD Lp(a) HORIZON trial (NCT04023552): ongoing
Olpasiran (AMG890)	healthy individuals [48] **Dosing**: single dose of 3, 9, 30, 75 and 225 mg of olpasiran **Baseline to day 43–71**: Lp(a) reduction: 71 to 97%	patients with established ASCVD [49] OCEAN(a)-DOSE trial **Dosing:** subcutaneous administration of olpasiran (10 mg every 12 weeks, 75 mg every 12 weeks, 225 mg every 12 weeks, or 225 mg every 24 weeks) or placebo **Baseline to 36 weeks:** placebo-adjusted mean % changes in Lp(a): −70.5% (95%CI, −75.1 to −65.9) in the 10-mg group, −97.4% (95%CI, −102.0 to −92.8) in the 75-mg dose group, −101.1% (95%CI, −105.8 to −96.5) in the 225-mg dose group given every 12 weeks, and −100.5% (95%CI, −105.2 to −95.8) in the 225-mg dose administered every 24 weeks placebo-adjusted percent change in LDL-C: −23.7% (95%CI, −35.3 to −12.2) in the 10-mg group, −22.6% (95%CI, −34.1 to −11.0) in the 75-mg dose group, −23.1% (95%CI, −34.8 to −11.4) in the 225-mg dose group given every 12 weeks, and −24.8% (95%CI, −36.5 to −13.0) in the 225-mg dose administered every 24 weeks placebo-adjusted percent change in apoB concentration: −18.9% (95%CI, −26.3 to −11.5) in the 10-mg group, −16.7% (95%CI, −24.1 to −9.3) in the 75-mg dose group, −17.6% (95%CI, −25.1 to −10.1) in the 225-mg dose group given every 12 weeks, and −18.8% (95%CI, −26.3 to −11.2) in the 225-mg dose administered every 24 weeks placebo-adjusted mean % change in OxPL-apoB from baseline: −51.6% (95%CI, −64.9% to −38.2%) for the 10-mg Q12W dose, −89.7% (95%CI, −103.0% to −76.4%) for the 75-mg Q12W dose, −92.3% (95%CI, −105.6% to −78.9%) for the 225-mg Q12W dose, and −93.7% (95%CI, −107.1% to −80.3%) for the Q24W dose **Baseline to 48 weeks:** placebo-adjusted mean % change in Lp(a): −68.5% (95%CI, −74.3 to −62.7) in the 10-mg group, −96.1% (95%CI, −101.9 to −90.3) in the 75-mg dose group, −100.9% (95%CI, −106.7 to −95.0) in the 225-mg dose group given every 12 weeks, and −85.9% (95%CI, −91.8 to −80.1) in the 225-mg dose administered every 24 weeks placebo-adjusted mean % change in OxPL-apoB from baseline: −50.8% (95%CI, −64.9% to −38.2%) for the 10-mg Q12W dose, −100.2% (95%CI, −103.0% to −76.4%) for the 75-mg Q12W dose, −104.7% (95%CI, −105.6% to −78.9%) for the 225-mg Q12W dose, and −85.8% (95%CI, −107.1% to −80.3%) for the Q24W dose	patients with established ASCVD OCEAN(a)-Outcomes trial (NCT05581303): ongoing
Zerlasiran (SLN360)	healthy individuals [52] **Dosing**: single doses of zerlasiran administered subcutaneously at 30 mg, 100 mg, 300 mg, or 600 mg versus placebo **Baseline to day 150**: maximal median changes in Lp(a) levels: −20 (IQR, −61 to 3) nmol/L in the placebo group, −89 (IQR, −119 to −61) nmol/L in the 30-mg group, −185 (IQR, −226 to −163) nmol/L in the 100-mg group, −268 (IQR, −292 to −189) nmol/L in the 300-mg group, and −227 (IQR, −270 to −174) nmol/L in the 600-mg group maximal median percentage changes in Lp(a) levels: −10% (IQR, −16% to 1%) in the placebo group, −46% (IQR, −64% to −40%) in the 30-mg group, −86% (IQR, −92% to −82%) in the 100-mg group, −96% (IQR, −98% to −89%) in the 300-mg group, and −98% (IQR, −98% to −97%) in the 600-mg group maximum reduction in mean apoB level: 24% at 30 days after the 600-mg dose and 19% measured 14 days after the 300-mg dose maximum reduction in mean oxidized LDL level: 20% in the 600-mg dose group and 11% in the 300-mg dose group maximum LDL-C reduction: 26% in the 600-mg dose group healthy individuals and patients with established ASCVD [47] **Dosing**: 1 year follow-up for participants who received a single dose of zerlasiran 300 mg or 600 mg + individuals who received 2 doses of placebo, zerlasiran 200 mg at a 4-week interval or 300 mg or 450 mg at an 8-week interval **Baseline to 365 days:** After a single dose, maximal median Lp(a) changes: +14% (IQR, +13% to +15%) for the placebo group, −30% (IQR, −51% to −18%) for the 300 mg of zerlasiran group, and −29% (IQR, −39% to −7%) for the 600-mg dose group After 2 doses, maximal median Lp(a) changes: +7% (IQR, −4% to +21%) for the placebo group, −97% (IQR, −98% to −95%) for the 200 mg of zerlasiran group, −98% (IQR, −99% to −97%) for the 300 mg of zerlasiran group, and −99% (IQR, −99% to −98%) for the 450 mg of zerlasiran group maximal median change in LDL-C levels: +17% (IQR, −2% to +29%) observed at 150 days after placebo administration, −35% (IQR, −45% to −26%) at 60 days for the 200-mg dose group, −47% (IQR, −64% to −12%) at 30 days for the 300-mg dose group, and −28% (IQR, −38% to −26%) at 90 days for the 450-mg dose group. maximal median change in apoB: +12% (IQR, +2% to +17%) at 150 days after placebo administration, −26% (IQR, −35% to −8%) at 43 days for the 200-mg dose group, −28% (IQR, −37% to −21%) at 90 days for the 300-mg dose group, and −23% (IQR, −34% to −22%) at 90 days for the 450-mg dose group	patients with established ASCVD [54] ALPACAR-360 trial **Dosing**: subcutaneous administration of placebo (3 doses every 16 weeks or 2 doses every 24 weeks) or zerlasiran (2 doses of 450 mg every 24 weeks, 3 doses of 300 mg every 16 weeks or 2 doses of 300 mg every 24 weeks) **Baseline to 36 weeks:** Median percent change in Lp(a) levels: −94.5% (IQR, −97.3% to −84.2%) for the 450 mg every 24 weeks group, −96.4% (IQR, −97.7% to −92.3%) for the 300 mg every 16 weeks group, and −90.0% (IQR, −93.7% to −81.3%) for the 300 mg every 24 weeks group. placebo-adjusted time-averaged percent change from baseline in Lp(a): −85.6% (95%CI, −90.9% to −80.3%) for the 450 mg every 24 weeks group, −82.8% (95%CI, −88.2% to −77.4%) for the 300 mg every 16 weeks group, and −81.3% (95%CI, −86.7% to −76.0%) for the 300 mg every 24 weeks group. placebo-adjusted time-averaged percent change from baseline in LDL-C: −25.1% (95%CI, −46.9% to −3.3%) for the 450 mg every 24 weeks group, −31.9% (95%CI, −54.1% to −9.7%) for 300 mg every 16 weeks group and −29.7% (95%CI, −51.6% to −7.8%) for the 300 mg every 24 weeks group placebo-adjusted time-averaged percent change from baseline in apoB: −15.0% (95%CI, −20.1% to −9.8%) for the 450 mg every 24 weeks group, −13.3% (95%CI, −18.6% to −8.1%) for 300 mg every 16 weeks group and −9.9% (95%CI, −15.0% to −4.7%) for the 300 mg every 24 weeks group **Baseline to 48 weeks:** placebo-adjusted time-averaged percent change from baseline in Lp(a): −83.0% (95%CI, −88.4% to −77.5%) for the 450 mg every 24 weeks group, −83.1% (95%CI, −88.7% to −77.6%) for the 300 mg every 16 weeks group, and −78.7% (95%CI, −84.2% to −73.2%) for the 300 mg every 24 weeks group. placebo-adjusted time-averaged percent change from baseline in LDL-C: −26.0% (95%CI, −44.7% to −7.2%) for the 450 mg every 24 weeks group, −29.8% (95%CI, −48.9% to −10.8%) for 300 mg every 16 weeks group and −27.4% (95%CI, −46.2% to −8.6%) for the 300 mg every 24 weeks group placebo-adjusted time-averaged percent change from baseline in apoB: −14.0% (95%CI, −19.2% to −8.8%) for the 450 mg every 24 weeks group, −12.4% (95%CI, −17.6% to −7.1%) for 300 mg every 16 weeks group and −8.6% (95%CI, −13.9% to −3.4%) for the 300 mg every 24 weeks group **Baseline to 60 weeks:** placebo-adjusted time-averaged percent change from baseline in Lp(a): −77.1% (95%CI, −83.1% to −71.2%) for the 450 mg every 24 weeks group, −79.2% (95%CI, −85.3% to −73.1%) for the 300 mg every 16 weeks group, and −71.8% (95%CI, −77.8% to −65.8%) for the 300 mg every 24 weeks group. placebo-adjusted time-averaged percent change from baseline in LDL-C: −24.1% (95%CI, −43.9% to −4.2%) for the 450 mg every 24 weeks group, −28.7% (95%CI, −48.9% to −8.5%) for 300 mg every 16 weeks group and −26.2% (95%CI, −46.1% to −6.2%) for the 300 mg every 24 weeks group placebo-adjusted time-averaged percent change from baseline in apoB: −12.6% (95%CI, −18.0% to −7.2%) for the 450 mg every 24 weeks group, −11.3% (95%CI, −16.8% to −5.7%) for 300 mg every 16 weeks group and −7.2% (95%CI, −12.6% to −1.7%) for the 300 mg every 24 weeks group	No trial
Lepodisiran (LY3819469)	healthy individuals [55] **Dosing**: a single dose of lepodisiran (4 mg, 12 mg, 32 mg, 96 mg, 304 mg, or 608 mg) or placebo administered subcutaneously **Baseline to 48 weeks**: maximal median change in Lp(a) concentration: −5% (IQR, −16% to 11%) in the placebo group, −41% (IQR, −47% to −20%) in the 4-mg group, −59% (IQR, −66% to −53%) in the 12-mg group, −76% (IQR, −76% to −75%) in the 32-mg group, −90% (IQR, −94% to −85%) in the 96-mg group, −96% (IQR, −98% to −95%) in the 304-mg group, and −97% (IQR, −98% to −96%) in the 608-mg dose group.	healthy individuals (Phase II) [56] **Dosing**: lepodisiran at a dose of 16, 96, or 400 mg at baseline and at day 180, lepodisiran at a dose of 400 mg at baseline and placebo at day 180, or placebo at baseline and at day 180 **Baseline to 180 days**: placebo-adjusted time-averaged % change in Lp(a) levels: −40.8% (95%CI, −55.8 to −20.6), −75.2 % (95%CI, −80.4 to −68.5) and −93.9% (95%CI, −95.1 to −92.5) in the 16-mg, 96-mg lepodosiran group, and in the pooled 400-mg groups, respectively	patients with established ASCVD ACCLAIM-Lp(a) (NCT06292013): ongoing
Muvalaplin (LY3473329)	healthy individuals [60] **Dosing**: single ascending dose of muvalaplin from 1 mg to 800 mg or multiple ascending muvalaplin doses ranging from 30 mg to 800 mg, or placebo, administered daily **Baseline to 14 days**: placebo-controlled Lp(a) reduction: 63% to 65% at doses of 100 mg or more	patients with established ASCVD, familial hypercholesterolemia, diabetes KRAKEN trial [62] **Dosing**: orally administered muvalaplin at dosages of 10 mg, 60 mg or 240 mg daily, or placebo **Baseline to 12 weeks**: placebo-adjusted Lp(a) reductions: 47.6% (95%CI, 35.1% to 57.7%) in the 10-mg group, 81.7% (95%CI, 78.1% to 84.6%) in the 60-mg group and 85.8% (95%CI, 83.1% to 88.0%) in the 240-mg group, using an intact lipoprotein(a) assay and 40.4% (95%CI, 28.3% to 50.5%) in the 10-mg group, 70.0% (95%CI, 65.0% to 74.2%) in the 60-mg group and 68.9% (95%CI, 63.8% to 73.3%) in the 240-mg group, using an apolipoprotein(a)-based assay. dose-dependent placebo-adjusted apoB changes: −8.9% (95%CI, −18.8% to 2.2%), −13.1% (95%CI, −20.9% to −4.4%), and −16.1% (95%CI, −23.7% to −7.8%) at 10 mg, 60 mg, and 240 mg group, respectively	No trial

## Data Availability

No new data were created or analyzed in this study. Data sharing is not applicable to this article.
